# Does the digital economy improve female employment? A cross-country panel data analysis

**DOI:** 10.1016/j.heliyon.2024.e33535

**Published:** 2024-06-27

**Authors:** Riaz Ahmad, Fatima Sharif, Sareer Ahmad, Azeem Gul, Zhainagul Abdirasulova Abdirasulovna

**Affiliations:** a School of Business, Qilu Institute of Technology, China; b School of Economics, Quaid-I-Azam University, Islamabad, Pakistan; c National University of Modern Languages, Islamabad and PhD Candidate at Quaid-i-Azam University in Area Study Centre for Africa, USA; d Osh State University, International Medical Faculty, Osh Kyrgyzstan

**Keywords:** Digital economy, Female employment, Cross-sectional dependence, Cointegration, PARDL

## Abstract

The digital economy has had an impact on the female employment rate over time. Currently, the researchers are more interested in investigating the impacts of the digital economy by focusing on its various aspects of female employment. The current study is motivated by this renewed interest to investigate the impact of the digital economy on female employment rates in Asian Developing Countries from 1990 to 2021. The digital economy is measured by several indicators such as fixed telephone subscriptions, fixed broadband subscriptions, mobile phone subscriptions, secure internet servers, and internet users. The Panel Autoregressive Distributed Lag (PARDL) model is used for analysis that reveals a positive relationship between female employment rates and the digital economy in both the short and long run. The control variables/factors, Education and GDP, also showed positive relationships with female employment. We suggest that governments prioritize funding for digital infrastructure and encourage fair access to technology, especially for women, based on our study. Furthermore, the positive effects of the digital economy on female employment can be strengthened through focused policy interventions, such as offering financial incentives to companies that hire and train women in digital skills. By utilizing these tactics, policymakers can guarantee that women are prepared to take advantage of the chances brought about by the quickly changing digital landscape, promoting gender equality and inclusive economic growth throughout Asian developing nations.

## 1 Introduction

Equitable work opportunities for women are a global subject matter. Gender disparity in the workforce has long been a problem on a global scale. The main obstacles that women encounter in the labor market include low employment rates, an unfavorable employment structure, and few prospects for career advancement, according to research by the International Labour Organization [1]. Governments and academics have been paying more attention to the issue of female labor market participation declining during the past 30 years in many different countries [2]. Digitization currently characterizes the global economy. These days, the internet, cloud computing, big data, artificial intelligence, and other next-generation information technologies power the digital economy, which permeates every aspect of society and business. Digital technology and the economy are closely linked, and the digital economy is growing quickly at a time when the world's economic growth is decreasing and the technological revolution is propelling the globe into a new phase of growth [3].

The digital economy opens a world of economic opportunities for women, regardless of whether they are rejected from the mainstream workforce [4]. The digital or web economy is defined as economic activities driven by the internet and electronic technologies. Online platforms, e-commerce, digital services, and other digital technologies have revolutionized the way businesses act and consumers engage with them. There is an increasing interest in finding how the digital economy affects employment, particularly for women. Traditionally, women have experienced hurdles in accessing the labor market, especially in developing nations, where gender inequities are more frequent [5]. Nonetheless, the growth of the digital economy has offered new chances for women to enter the labor field, notably in technology and other digital-related industries. As a result, there is considerable interest in investigating whether the digital economy has increased female employment prospects and what variables may be contributing to these developments [6]. In general, women's employment participation in South Asian countries has been low and largely unchanged since 2001. In particular, the gender employment gap is more noticeable in middle age groups. Female employment is higher in rural areas than in urban ones. The largest share of female employment is in agriculture, though this is slowly changing in some countries. Finally, women with mid-level education tend to have lower employment rates than those with both lower and higher education. These findings are reported in the paper by Ref. [7]. [2] investigate the impact of the digital economy on female employment in China. The empirical findings imply that female employment is greatly encouraged by the digital economy. According to the estimates, this association is consistent with our theoretical analysis because the digital economy emphasizes the need for female-preference occupations by creating opportunities for women to use digital technology, forming egalitarian gender perspectives, and increasing labor demand.

Every country is seeking solutions to achieve gradual and long-term development. The development of the country's GDP per capita is heavily reliant on technical innovation. Countries with high levels of innovation and technology are often more competitive and perform better economically [8,9]. Several studies on ICT (Information and Communications Technology) influence on innovation have found that the use of Information and Communications Technologies, particularly the Internet facilitates the diffusion of tacit and codified knowledge as well as the development of new products, processes, businesses, and intercompany collaboration [10]. Hence, Information and Communications Technologies encourage as well as promote the potential for innovation by developing information networks that enable knowledge to be disseminated [11]. [12] investigated how gender wage rate discrimination is affected by the iron-out effect of the digital economy during working hours. They discovered that men's wage rates are much higher than women's, with the discriminatory effect of working hours accounting for 14%–19 % of the overall difference. The digital economy has the potential to mitigate gender discrimination in work hours by increasing women's return on employment.

Furthermore, they enable the identification of new sources of invention, the development of research and creativity, and the lack of time to market [13]. ICT that recognizes new client needs, new production and logistics processes, and new customer segments enables strategic innovation [14]. The study investigated the effect of male violence on labor force participation among 824 women who took part in a random residential survey in a low-income Chicago neighborhood. In the year preceding the survey, eighteen percent had been physically assaulted, while 11.9 percent of the total had been subjected to more serious violence. Throughout their adult lives, 40.3 percent of respondents had seen coercive and threatening behavior, and 28.4 percent of the surveyed had witnessed a criminal assault. According to the findings, Women who experienced male violence were just as likely as those not currently employed to have a history of unemployment, health problems, and higher rates of welfare receipt [15]. There are additional cognitive irregular activities that can be mechanized. For example, chronic disease and cancer treatment evaluations have been partially automated using data analytic on large volumes of medical records for bench marking and pattern recognition [16].

The importance of female employment in the digital economy cannot be emphasized. Women's labor-force involvement is critical for economic growth and development, and the digital economy provides an enormous opportunity for women to participate in the labor force in meaningful ways. To begin with, female employment in the digital economy can contribute to closing the gender gap in technology and creativity. We can assist in creating a more diverse and inclusive workforce by encouraging more women to participate in Science, Technology, Engineering, and Mathematics (STEM) Disciplines, which can lead to increased innovation and creativity. Second, the digital economy allows for greater flexibility in employment arrangements, which can be advantageous for women who need to balance work and family commitments. Remote work and flexible hours can help women better manage their time and lessen the stress of unpaid care work, which disproportionately affects women. Finally, employing women in the digital economy can help reduce the gender pay gap. In many industries, women are paid less than men for doing the same work, but the digital economy provides more transparency and can help to level the playing field by offering equal pay for equal work. Overall, the value of female labor in the digital economy cannot be overstated. We can promote gender equality, foster economic growth and innovation, and create a more inclusive and diverse workforce by encouraging more women to participate in this sector [17]. [18] Explore the relationship between Female employment and the digital economy in India. The data clearly shows the pitifully low skill levels of women. Notably, improving skill development in line with the demands of the rising market including digital literacy will significantly increase the number of work prospects available to women [19]. explore the association between digital technology, digital innovation, and ICT companies in Pakistan. They collected 396 samples to analyze the data. The results highlight the beneficial and noteworthy effects that digital capability, digital orientation, and digital transformation have on DI and FP. Furthermore, there is a strong and favorable correlation between FP and DI. Lastly, the relationships between DC, DO, DT and FP are mediated by DI.

The geographical and economic conditions of each country's digital economy have a varied impact. These small and medium enterprises (SMEs) companies employ about 67 % of all employees. They generate 58 % of the total turnover in the European Union (EU) and are the backbone of the economy [20]. The digital or web economy provides the potential for stronger relationships and networks with employees and employers. While the major driver of a firm's adoption of digital or new technology can be predicted as increased labor efficiency, companies may have other objectives – client demands or better competition and this also affects employment [21]. A recent issue of the impact of ICT on overall employment has become a major source of controversy among both academics and policymakers. According to Ref. [22], the revolution of ICT is putting US jobs in danger due to computers' propensity to replace humans in everyday work.

Cultural, sociological, and institutional elements are important in determining how the digital economy and female employment interact in developing Asian nations: **Cultural factors**: Women's participation in the digital economy, access to education, and work prospects can all be impacted by cultural norms and traditions. Patriarchal civilizations, for instance, might place a higher value on men's education and employment, which would restrict the participation of women in the digital industries. Cultural perceptions about women's duties and responsibilities may influence whether they choose to work in technology or entrepreneurship. **Social Factors**: Women's participation in the digital economy can be influenced by social networks and support systems. Women can be empowered to pursue digital careers and entrepreneurship by having access to peer networks, mentors, and role models. On the other hand, social obstacles including discrimination, gender stereotypes, and cultural expectations could prevent women from entering the digital workforce. **Institutional Factors:** The opportunities and problems facing women in the digital economy are shaped by government policies, regulatory frameworks, and institutional support systems. Policies that support entrepreneurship, education access, and gender equality can foster a climate that is favorable to women working in digital industries. On the other hand, structural impediments including restricted financial resources, inadequate childcare assistance, and biased labor regulations could hinder women's involvement and progress in the digital workforce. In short, implementing successful initiatives to support female employment in the digital economy in Asian developing nations requires an awareness of the interplay of cultural, social, and institutional elements. Policymakers, companies, and civil society may promote more inclusive and equal chances for women to prosper in the digital era by addressing these factors.

It becomes an important research agenda to conduct a thorough study of the nexus between female employment rates and the digital economy in Asian developing countries. Bangladesh, Cambodia, China, India, Indonesia, Lao People's Democratic Republic, Malaysia, Mongolia, Nepal, Pakistan, Philippines, Sri Lanka, Tajikistan, Thailand, Turkmenistan, and Vietnam are among the countries involved. This study has the potential to provide new opportunities for women to work from home and earn a living. Constructing an index to assess the scope and current situation of female employment rates in developing economies can provide valuable insights into the challenges and opportunities faced by women in this area. In the absence of existing research covering this issue in developing countries, this study identifies it as a possible research gap and contributes to this field of study. The discussions and findings made in this study will help identify ways to improve female employment and address gender gaps in the workforce by exploring the potential benefits of the digital economy for women. Thus, it provides practical and policy implications to regulators, policymakers, and other relevant stakeholders. The available scarce literature tested the digital economy's impact on different economic variables; we are inspired by this renewed interest to research the digital or web economy's influence on employment, especially among women in Asian developing countries.

Under this circumstance, this study undertakes two research questions, namely, (a) What is the impact of digital or web economy on women's employment in Asian developing countries? And (b) can developing countries empower females by upgrading digital infrastructure? The study employs a quantitative research strategy to investigate the study's findings by analyzing panel data from 16 Asian developing countries for 32 years. The study considers female employment as a dependent variable and the digital economy as an independent variable (fixed telephone subscriptions, fixed broadband subscriptions, mobile phone subscriptions, secure internet servers, and internet users as proxies), with GDP as well as education as control variables. The PARDL method is used to estimate the likely short and long-run relationship between chosen variables. The insights drawn from the focus on Asian developing countries may have limited applicability to other regions or economic situations due to differences in socioeconomic conditions, cultural norms, and levels of technical innovation. In various socioeconomic contexts, such as developed countries or developing regions, the relationship between the digital economy and female employment may be influenced by factors such as higher levels of digital infrastructure, different gender norms, and more established labour market institutions. For example, in rich economies, the digital economy may be more interconnected, and female employment may confront different constraints than in underdeveloped nations. As a result, while the study provides useful insights, caution should be given when extrapolating these findings to different contexts, as the interplay between the digital economy and female employment can differ greatly depending on local socioeconomic conditions. The findings show that the digital economy can greatly boost female employment, contributing to overall socioeconomic growth by raising household incomes and lowering poverty. Promoting digital access and skills for women can also help to advance gender equality by increasing participation in the labour force and decision-making processes, which is critical for inclusive and sustainable development.

The following is how the article's body is organized. Chapter two contains a literature review, while the Third chapter contains the research methodology. The fourth chapter concludes with a conclusion and debate, while the Fifth chapter ends with a conclusion and policy recommendations.

## 2 Literature review

Studies on the digital economy and its impact have received significant attention in recent years. Existing literature gives a rich archival record in this regard. Keeping our research objectives under consideration, we have accumulated a summary of existing research across our major themes i.e., digital economy and female employment. It provides a glimpse of the existing body of knowledge on the selected areas and also helps us to identify potential research gaps.

Governments and academics have been paying more attention to the issue of female labor market participation declining during the past 30 years in many different countries [2]. An increasing collection of literature investigates the digital or web economy's impact on female employment. According to available studies, the digital or web economy has the potential to boost female employment prospects by allowing for flexible work schedules and reducing gender-based discrimination. Some studies, on the other hand, highlight the risks of automation and the potential for the digital economy to intensify current gender inequities.

Achieving economic growth and sustainable development necessitates gender equality and female economic engagement (International Labour Organization [23]. Nevertheless, because of unfavorable social norms, gender differences in unpaid domestic and care giving work, discriminatory laws and insufficient legal protection, and unequal access to digital, financial, and property assets, the gender gap in the labor force participation rate henceforth referred to as the gender gap remains significant. When taken as a whole, these disparities put women at a disadvantage on the social and economic fronts [23]. To use digital technology tools to solve gender equality issues in the Sustainable Development Goals, it is important to look at how digitization has affected the labor market [24]. examine the attitudes of women in Russia towards distance employment, also known as remote work or telecommuting. The research conducted a sociological survey to gather data on this topic and found that distance employment is increasingly becoming popular among women in Russia. Many women perceive distance work positively and see it to improve their quality of life in terms of social, cultural, family, and reproductive factors. The study [25] also found that more than one-third of women looking for work prefer the remote format to traditional employment. Furthermore [26], investigate the role of online freelancing in boosting women's empowerment by using Pakistan's transitional economy. It specifically assesses the barriers to employment that women meet, such as financial independence, online freelancing, household autonomy, and professional identity. It continues to expand the concept of freelancing and the chances available to Pakistani youth in general and notably for women. The research includes a primary survey as well as an online survey. The findings of the study reveal the ability to influence freelancers. Women's empowerment and participation contribute to Pakistan's digital or web economy's growth. There are also limitations, such as a fear of failure and a lack of knowledge about remote work. This project can help with the execution of some of these measures, considering the limited research on Pakistan's economic transition owing to freelancing, its potential, and activity gaps.

Furthermore, the digitization of commerce and global value chains has significantly altered the globalization environment, with significant ramifications for women in the workforce and as merchants [27]. The gender gap may theoretically get smaller or larger depending on how globalization and digitization interact. First, by lowering transaction and trade costs, the adoption of digital technologies can increase international trade and investment activity. Because non-agricultural industries often pay higher incomes than agricultural sectors, this tends to give women access to a greater variety of career possibilities and raise the proportion of working women in these sectors [28]. Thus, the gender gap will close as a result of women's rising income. Second, a faster growth rate in online tradable services like financial, educational, and customer services is associated with a greater use of digital technology [27,29,30]. This would further encourage the growth of this business by drawing additional investment to the developing service sectors.

According to Ref. [31], the Economic capital was able to create 3.4 million new jobs each year (1.5 million directly and 3.4 million indirectly) and boost the rate of employment to the desired value. Because many women lack these abilities, the government has set up a program to educate women about such facilities. It is free and allows you to improve your technical abilities and productivity. This works when 15,000 persons were recognized as freelancers and another 500,000 expressed interest in participating in the learning [32]. As an outcome, women's conditions in terms of career opportunities have greatly improved. According to Ref. [33] a new report, 25 % of freelancers are females who outperform males in the country. Their performance is $2 higher than that of males. Furthermore, the government has been experimenting with policies to encourage current IT businesses to learn and gain experience, resulting in improved remuneration for women in such facilities. As a result of the rapid proliferation of digital technology, Pakistan has decided to boost its commercial viability through outsourcing and freelance work [34]. [35]. used a sample of 42 sub-Saharan African nations from 2004 to 2014 to investigate the effect of ICT on female labor market participation. They determined criteria for ICTs to modify inequality to raise female labor force participation in their study [36]. used data from Jordan spanning the years 2010–2016 to further substantiate the promotion effect of internet adoption on female labor force participation.

In terms of total development, urban and rural China's digital economies have exhibited consistent growth, but rural areas continue to lag behind urban areas. The degree of development of digital buildings varies due to differences in economic development between urban and rural areas. The digital divide is due to regional differences in the level of development of the digital economy. There are two types of digital divides: first-class and second-class, with notable differences in infrastructural development and data and information collection, filtering, processing, and utilization [37]. Rural China has a low overall education level, as well as a shortage of knowledge about digital or web technology such as the World Wide Web, keeping them vulnerable to expanding Internet restrictions. Farmers' low level of use of digital technology has led to a more serious problem of the “digital divide”, which hinders the improvement of labor skills and wealth acquisition among rural residents and makes them more successful in their means of profit, and prevents spreading [38]. The authors [39] have discussed the concept of technological developments, which are inventions that initiate new cycles of development of productive force and lead to the transformation of socio-economic formations. PCs, internet, broadband, global positioning systems, cellular devices, digital technology, robots, radio-frequency identification tags, renewable energy, 3D printers, artificial intelligence, and cloud technologies have been identified as disruptive technologies in the growth of the Internet of Things and by 2024, 37 billion devices will be connected to the ICT. Positive as well as negative effects of disruptive technologies and their economic implications are also discussed in this paper as well as the dilemma of innovators with two disruptive technology principles i.e., creativity and destruction. The study estimates the effects of important variables on global GDP per capita and finds that all factors i.e., growth in subscribers of cellular devices, increases in longevity, fixed subscribers of telephone and energy use, gross capital formation, etc. have a favorable impact on economic productivity [40].

According to the literature, the digital economy has the potential to boost female career opportunities, but it also brings with it risks and obstacles, particularly in terms of automation and gender discrimination. Access to education and training, encouragement for entrepreneurship, and efforts to remove gender biases in technology design and implementation are all needed to ensure that women may fully benefit from the opportunities given by the digital economy.

There is still a dearth of research on how the digital affects female employment, especially when it comes to developing Asian nations. While some studies, like those conducted in Turkey by Ref. [41] and in South Asia by Ref. [42], offer insightful information, there is a significant lack of research that focuses especially on the relationship between the digital economy and the employment rates of women in emerging Asian countries. By investigating how the digital economy affects female employment rates in Asian emerging nations, our research seeks to close this important gap. In contrast to previous research that mainly utilizes the ICT index, we utilize novel proxy variables to gauge the digital economy, providing a novel viewpoint on this intricate correlation.

By concentrating on female workers who have gotten comparatively less attention in this area, our research adds to the body of literature already available on digitization and employment. Since the literature review includes multiple studies, a more in-depth comparison with existing research would help readers grasp this study's unique contributions, emphasizing its specific findings and breakthroughs in the context of the existing body of knowledge. Future studies should examine the effects of the digital economy on female employment across industries and whether digitization leads to the polarization of employment based on gender in different industries.

## 3 Data and methodology

**Data:** The research aims to emphasize the relationship between the digital economy and women's employment in 16 Asian developing countries including Bangladesh, Cambodia, China, India, Indonesia, Lao People's Democratic Republic, Malaysia, Mongolia, Nepal, Pakistan, Philippines, Sri Lanka, Tajikistan, Thailand, Turkmenistan, and Vietnam. This quantitative analysis employs data from Asian developing countries published by the World Bank in its WDI (World Development Indicators) report over 32 years, from 1990 to 2021. The dependent variable is the female employment rate, while the explanatory variable is the digital economy, which is represented by fixed telephone subscriptions, fixed broadband subscriptions, mobile phone users, internet subscribers, and internet security servers. The digital economy was measured using specific measures, like internet users and broadband subscriptions, which show the level of digital connection and technological infrastructure in a given location. These indicators are important when discussing female employment since they indicate that women can take advantage of remote work, online entrepreneurship, and digital skill development. increasing access to resources and employment opportunities for women may be shown by higher levels of internet usage and broadband subscriptions. This could result in increasing female engagement in the workforce and empowerment through digital employment chances. Also, GDP and education level are included as control variables in the model. The GDP and education levels can have a big impact on how the digital economy and female employment interact: education: Women who have completed higher education are more likely to be able to use digital technology efficiently, which will increase their engagement in labor and the digital economy. Women who have an education are better equipped to take advantage of digital platforms for entrepreneurship, job growth, and skill advancement. These indicators were chosen because they directly reflect access to and use of digital technology, which are essential components of the digital economy. Increased internet users and broadband subscriptions allow for greater participation in online work, digital entrepreneurship, and access to information, all of which can have a significant impact on female employment by creating new opportunities and lowering barriers that women have traditionally faced in the workplace. GDP: A higher GDP denotes more comprehensive economic development, which may foster an atmosphere that is conducive to the expansion of the digital economy. Increased innovation, technology adoption, and investment in digital infrastructure can all result from higher GDPs, and these factors can boost the number of jobs available to women in the digital industry and allied fields.

In conclusion, by influencing digital literacy, skill development, and general economic conditions, education and GDP can have an impact on the relationship between the digital economy and female employment. They might also engage in interactions and act as a mediator in this relationship by making digital technology more accessible and creating an atmosphere that is supportive of female employment. Education and GDP are important control variables because they influence the relationship between the digital economy and female employment. Higher education levels can improve digital abilities, increasing women's competitiveness in the digital labour market. GDP growth signals economic prosperity, which may increase job prospects, notably in the digital sector. Furthermore, there may be interactions in which an educated female workforce in a growing economy benefits more from technological breakthroughs, underlining the potential mediating impacts of education and economic growth on female employment. In Table 1, variables are listed which are used in this research (see Table 2).

**Table 1:** variable description and data source.

Variable Name	Representation	Description	Source
• Female Employment rate	FE	%offemaleemployment(self−emplyed)	World bank
• Subscriptions for Fixed Telephones (Per 100 People)	FTS	Usersoftelephoneline	WDI
• Subscriptions for fixed broadband (Per 100 People)	FBS	Wireless technology users or Public internet	WDI
• Subscriptions for Mobile phones(Per 100 People)	MCS	Mobilephonesusebyindividual	WDI
• Secure Internet Servers (PerMillion People)	SIS	Secure network under government supervision	WDI
• Individuals using the Internet(Percentage of Pop.)	IUI	UseofInternetperperson	WDI
• Gross Domestic Product	Ln(GDP)	GDP(ConstantUSdolar)	WDI
• Education	EDU	Schoolenrollment,tertiary(%gross)	WDI

**Table 2:** Descriptive analysis.

Variables	Mean	Med.	Max.	Min.	SD	Skew	Kurt.	JQ
FE	0.787	0.515	3.190	0.000	0.702	1.221	4.017	146.98
FTS	5.907	3.609	28.0	0.034	6.015	1.333	4.098	174.79
FBS	2.221	0.50	37.575	0.000	4.568	3.982	23.025	9753.4
SIS	123.8	0.5	7306.1	0.1865	623.6	8.059	77.642	122456.8
IUI	12.873	2.029	96.751	0.000	19.738	1.896	6.021	493.90
MCS	48.26	22.28	181.76	0.000	53.09	0.658	1.961	59.10
EDU	16.30	12.73	67.69	0.500	15.75	1.030	3.408	92.65
Ln(GDP)	24.47	24.85	30.50	0.500	3.250	−3.857	30.037	16601.05

### 3.1 Modeling framework

**Fig. 1:**
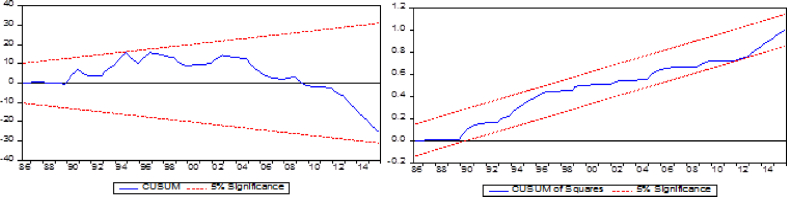
Sensitivity analysis CUSUM and CUSUMsq

The econometric model presented here is used to evaluate the digital or web economy's impact on female employment rates. Furthermore, gross domestic product and level of education are included as control variables. According to this, we have developed our modeling framework which is as figured (Fig. 1) below: The digital economy was measured using specific measures, like internet users and broadband subscriptions, which show the level of digital connection and technological infrastructure in a given location. These indicators are important when discussing female employment since they indicate that women can take advantage of remote work, online entrepreneurship, and digital skill development. increasing access to resources and employment opportunities for women may be shown by higher levels of internet usage and broadband subscriptions. This could result in increasing female engagement in the workforce and empowerment through digital employment chances. Also, GDP and education level are included as control variables in the model. The GDP and education levels can have a big impact on how the digital economy and female employment interact: education: Women who have completed higher education are more likely to be able to use digital technology efficiently, which will increase their engagement in labor and the digital economy. Women who have an education are better equipped to take advantage of digital platforms for entrepreneurship, job growth, and skill advancement.

**Figure:**
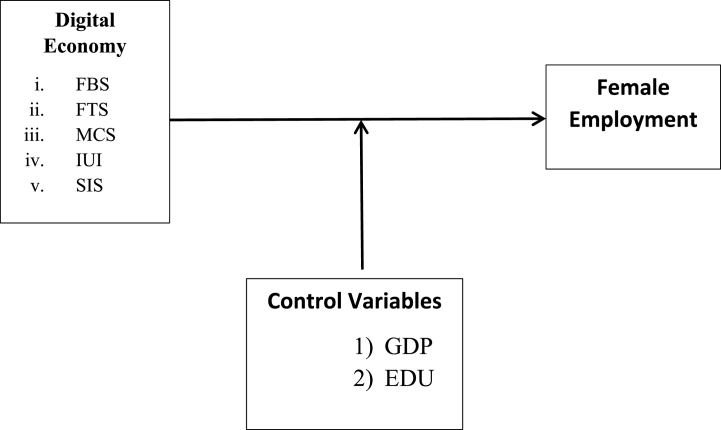

#### 3.1.1 Econometric model

We developed our econometric model for estimation by following [41], taking into consideration the data and econometric framework. In this study [41] investigates in Turkey, the digital economy's impact on women's employment. We improved the model based on available literature by combining critical considerations, thoughts, and explanations of the factors interactions. Our econometric model (female employment as a function of the digital economy) is shown in Equation (1) below:

Functional form:

$$\text{FE}=f\left({FTS}_{it},{FBS}_{it},{MCS}_{it},{IUI}_{it},{SIS}_{it},{GDP}_{it},{EDU}_{it}\right)\;\;\;\;\;\;\;\;\;\;\;\;\;\;\;\;\;\;\;\;\;\;\;\;\;\;(A)$$

Econometric Model:

Eq (1) $$\text{FE}it={\;\alpha }_{0}+\alpha_{1}\text{FTS}\;\text{it}+\alpha_{2}\text{FBSit}+\alpha_{3}\text{MCSit}+\alpha_{4}\text{IUIit}+\alpha_{5}\text{SISit}+\alpha_{6}\text{GDPit}+\alpha_{7}\text{EDUit}+\varepsilon_{it}$$

Where, FEₜ is the female employment rate of 16 Asian developing countries at time t, αₒ is the intercept, αᵢₜ indicates the coefficients of digital economy at time t which are proxies by five variables: fixed telephone subscription **(FTS)**, fixed broadband subscription **(FBS)**, mobile phone users **(MCS)**, internet subscribers **(IUI)**, and internet security servers **(SIS)**. Besides, GDPandEDU are control variables at t time. Lastly, εₜ denotes residual at ttime.

### 3.2 Econometric techniques

#### 3.2.1 Pesaran CD test (2004) for CSD (cross-sectional dependence)

In panel data, CSD appears commonly. Failure to account for CSD may result in inefficient and inaccurate regression estimates [43,44]. The presence of correlation or dependency between units (e.g., individuals, firms, countries) in a panel datasets is referred to as CSD in panel data. CSD can occur because of a variety of variables, including spillover effects, common shocks, and common unobserved components. Traditional panel data models that presume independence across units may produce skewed and inconsistent Estimates in the presence of CSD. As a result, CSD must be considered in panel data analysis. These test statistics are provided [45].

Eq (2) $$CD=\frac{\sqrt{2T}}{N\left(N-1\right)}(\sum_{i=1}^{N-1}\sum_{t=i+1}^{N}\beta ᵢₜ),N(0,1)$$

CCE (Pesaran's common correlated effects) estimator is one method for dealing with cross-sectional dependence (2006). The CCE estimator includes a common factor in the model that represents cross-sectional dependence and estimates model parameters using the generalized method of moments (GMM). Another method for addressing cross-sectional dependence is to apply SEM such as spatial error and SAR (spatial autoregressive). These models account for the geographical interactions among the units in the panel datasets and generate consistent parameter estimates [45]. It needs to be highlighted that the presence of CSD might have serious consequences for the validity of statistical inference in panel data analysis. As a result, before estimating panel data models, it is critical to assess for CSD (cross-sectional dependence). In panel data, [46]. test is well-known for detecting CSD. In panel model, when error components are associated across persons or units, this is referred to as CSD. CSD can cause biased and inefficient estimates, as well as influence the reliability of statistical inference.

#### 3.2.2 Test for slope heterogeneity

[47] introduced the Heterogeneity slope test. It is a popular panel data analysis tool for detecting heterogeneity in slope coefficients among individual units in a panel datasets. The estimate of Pooled OLS and Fixed effects model supports the test. The pooled OLS and fixed effect model should provide similar findings if the slope coefficients are homogeneous across individual units. The fixed-effects model, on the other hand, if the slope coefficients are heterogeneous, will provide different findings from the pooled OLS model. If the homogeneous slope coefficients null hypothesis is valid, test statistics should have a standard normal distribution. Depending on the study question, the slope heterogeneity test might be one-tailed or two-tailed [47]. The heterogeneity slope test has been frequently utilized in empirical investigations in domains ranging from economics to finance to management. It is a useful method to find heterogeneity in panel data and has been proved in Monte Carlo simulations to have good size and power qualities.

#### 3.2.3 Panel unit root test

Establish variable stationarity before performing panel data analysis and before using cointegration methods, decide the integration method to avoid a spurious regression problem. The Fisher ADF (Augmented Dickey-Fuller) test by Ref. [48], the Levin-Lin-Chu (LLC) test found by Ref. [49], and the Im-Pesaran-Shin (IPS) test introduced by Ref. [50] are performed as panel unit root tests. For each cross-section, the Fisher Augmented Dicky Fuller test combines the value of *P* from unit root testing. With *2n* degrees of freedom, it has a chi-sq. distribution as a non-parametric test. Where, in panel data, n is the number of firms. Following the [51] test-statistics is mentioned below:

Eq (3) $$\lambda =-2\sum_{i=1}^{n}\log_{e}\rho ᵢ$$

Where ρᵢ is represent the Augmented Dicky Fuller unit root test p-value for unit *i*. In eq. (4), the basic regression model is given which applied in both Levin Lin Chu and Im Pesaran Shin.

Eq (4) $$\Delta Yᵢₜ=\mu ᵢ+\rho Yᵢₜ₋+\sum_{k=1}^{m}\alpha ₖ\Delta Yᵢₜ₋ₖ+\delta ᵢₜ+\theta ₜ+\varepsilon ᵢₜ$$

Where Δ indicates the initial difference operator, m is the lag length, and unit specific fixed and time effects represented by μᵢ and θₜ. In addition, I both tests, null hypothesis that ρᵢ = 0 for all i, i.e. that all-time series are independent random walks is tested against the alternative hypothesis that ρᵢ = 0 for all i. The only difference between LLC and IPS tests is the underlying hypothesis description [51]. The current study uses a CIPS (Cross Sectional Augmented IPS) unit root test developed by Ref. [52]. to address the issue of interdependence. Therefore, use the CIPS unit root test of the second generation developed by Ref. [53].

The CIPS equation is given as follows:

Eq (5) $${\Delta y}_{\text{it}}=\alpha_{i}+\rho_{i}y_{\mathrm{it}-1}+\beta_{i}{ȳ}_{t-1}+\sum_{j=0}^{k}{ϒ}_{\text{ij}}\Delta {ȳ}_{\mathrm{it}-1}+\sum_{j=0}^{k}\delta_{\text{ij}}y_{\mathrm{it}-1}+{ԑ}_{\text{it}}$$

Where, αi, k, and t represent the determining term, the orderly variations, and the averages of the different categories respectively. The following statistics are obtained from CIPS using alternate Augmented Dickey Fuller (CADF) values:

Eq (6) $$\hat{\text{CIPS}}=N^{-1}\sum_{i=1}^{N}{\text{CDF}}_{i}$$

Here, CDF shows the cross-sectional augmented Dickey Fuller.

Throughout the cross sections, assuming a common unit root process, LLC defines a homogeneous alternative implying that all-time series are stationary, in which all ρᵢ are equal and significantly negative. However, IPS, like Fisher ADF, evaluates a less restricted heterogeneous alternative in which ri may differ and only a considerable portion of all-time series is stable, assuming that across cross section, there are individual unit root processes.

#### 3.2.4 Panel cointegration

According to Refs. [54,55]. the traditional methods of panel data, e.g., fixed effects, random effects, and instrumental variable estimators attempt to avoid handling the cross-sectional dependency of error components and result in incorrect findings. The cointegration technique developed by Ref. [56] is used in this study to examine the connection between female employment and the digital economy.

This method is particularly applicable and reliable when the error terms are cross-section dependent [57]. We employed cointegration approaches since the data showed cross-sectional dependency. The null hypothesis is assumed by the [56] cointegration test. The alternative hypothesis, which holds that cointegration exists, is refuted by the lack of cointegration among the variables. Group statistics look at cointegration within a cross-sectional segment, whereas panel statistics look at cointegration throughout the entire sample. The following is the [56] cointegration test equation:

$$WP=\frac{{WP}^{0}-\;N^{1/2}\;M_{WP}\left(s,l\right)\;}{\left(V_{WP}\left(s,l\right)\;\right)1/2}\;\to N\left(0,1\right)$$

(7) $$WG=\frac{{WG}^{0}-\;N^{1/2}\;M_{WG}\left(s,l\right)\;}{\left(V_{WG}\left(s,l\right)\;\right)1/2}\;\to N\left(0,1\right)$$

#### 3.2.5 Panel Auto Regressive Distributive Lag model

In panel data, Panel ARDL is a popular technique of econometric, which is used to estimate the relationship in long-run between variables. This technique combines the benefits of the ARDL model and panel data analysis, allowing for the estimate of long-run coefficients that are robust to heterogeneity, CSD and non-stationarity in the data. Panel Auto Regressive Distributive Lag model is designed to estimate the long run relation between the variables i.e., independent, and dependent while controlling for short-run dynamics and other relevant factors [58]. The model is represented in first-difference or level form depending on stationarity properties of variables involved. The long-run elasticity and the error correction term, which describe adjustment rate towards the long run equilibrium, are coefficients of interest.

In panel data, the Panel Auto Regressive Distributive Lag (PARDL) model is a prominent econometric technique for analyzing the association between independent variables and its lagged value as well as lagged values of dependent variables. Model is stated as follows [59].

Eq (8) yit=αi+δ1yit−1+∑j=1kβjxit−j+γ1zit−1+∑j=1mλjzit−j+εit

Here, yit represents dependent variable for individual i at time t, and αi shows the individual fixed effect, which captures unobserved variability among individuals. The coefficient of the lag dependent variable is δ1. At time t, xit-j represents the jth lagged value of the independent variable for individual i. jth lagged independent variable's coefficient is denoted by βj. At time t, zit-j denotes the jth lagged value of the control variable for individual i. Lagged control variable's coefficient is y1. The coefficient of the jth lagged control variable is denoted by j. εit is the error term respectively. Panel ARDL has been used in a variety of domains, including finance, international economics, environmental economics, and health economics. It has been useful in addressing a wide range of research issues, such as the effects of exchange rate volatility on trade flows, the causes of carbon emissions, and the relationship between health expenditure and economic development [60] created the method and demonstrated its utility through simulations and empirical applications in one of the main publications on panel ARDL. Since then, the approach has been extended and developed in a variety of ways, including accounting for cross-sectional dependency and structural discontinuities, as well as allowing for other types of non-stationarity in the variables.

The panel ARDL is defined as:

Eq (9) $${FE}_{it}=\beta_{0}+\beta_{1}{FTS}_{i,t-1}+\beta_{2}{FBS}_{i,t-1}+\beta_{3}{MCS}_{i,t-1}+\beta_{4}{IUI}_{i,t-1}+\beta_{5}{SIS}_{i,t-1}+\beta_{6}{GDP}_{i,t-1}+\beta_{7}{EDU}_{i,t-1}+\sum_{j=1}^{N1}\lambda_{ij}\Delta {FTS}_{i,t-j}+\sum_{j=0}^{N2}\gamma_{ij}\Delta {FBS}_{t-j}+\sum_{j=0}^{N3}\eta_{ij}\Delta {MCS}_{t-j}+\sum_{j=0}^{N4}\alpha_{ij}\Delta {IUI}_{t-j}+\sum_{j=0}^{N5}\phi_{ij}\Delta {SIS}_{t-j}+\sum_{j=0}^{N6}\delta_{ij}\Delta {GDP}_{t-j}+\sum_{j=0}^{N7}\theta_{ij}\Delta {EDU}_{t-j}+{ɛ}_{it}$$

Where *i* = 1,2, 3 … …. *N*; *t* = 1,2,3 … …, *T*.

Where ${FE}_{it}$ is the female employment over a period t for each cross-sectional unit i; FTS represents Subscriptions for Fixed Telephones and FBS indicates Subscriptions for fixed broadband respectively at period t; MCS represents Subscriptions for Mobile phones; SIS represents Secure Internet Servers, IUI represents Individuals using Internet, GDP represent Gross Domestic Product and EDU represents education respectively at time “t”; sample units are indicated by “i” whereas, the number of time periods is represented by “t”. It is probable to re-specify equation (5) to include the ECM (error correction term) as follows:

Eq (10) $${\Delta \text{FE}}_{\text{it}}=\delta_{i}\;v_{i,t-1}+\sum_{j=1}^{N1}\lambda_{ij}\Delta {FTS}_{i,t-j}+\sum_{j=0}^{N2}\gamma_{ij}\Delta {FBS}_{t-j}+\sum_{j=0}^{N3}\eta_{ij}\Delta {MCS}_{t-j}+\sum_{j=0}^{N4}\alpha_{ij}\Delta {IUI}_{t-j}+\sum_{j=0}^{N5}\phi_{ij}\Delta {SIS}_{t-j}+\sum_{j=0}^{N6}\delta_{ij}\Delta {GDP}_{t-j}+\sum_{j=0}^{N7}\theta_{ij}\Delta {EDU}_{t-j}+{ɛ}_{it}$$

*Where* $V_{i,t-1}$ = ${\text{FTS}}_{t,t-1}$ − $\varphi_{0i}$ − $\varphi_{1i}{\text{FBS}}_{t-1}$ − $\varphi_{2i}{\text{MCS}}_{t-1}$ − $\varphi_{3i}{I\text{UI}}_{t-1}$-$\varphi_{4i}{\text{SIS}}_{t-1}$-$\varphi_{5i}{\text{IUI}}_{t-1}$-$\varphi_{6i}{\text{GDP}}_{t-1}-\varphi_{7i}{\text{EDU}}_{t-1}$ is the Linear error-correction term for each unit and the parameter $\delta_{i}$ is the error correction speed of adjustment for each unit which is equalent to $\beta_{1i}$.The parameters $\varphi_{1i}$, $\varphi_{2i}$, $\varphi_{3i}$, and $\varphi_{4i}$ are computed as $-\frac{\beta_{0i}}{\beta_{1i}}$ , $-\frac{\beta_{2i}}{\beta_{1i}}$, $-\frac{\beta_{3i}}{\beta_{2i}}$, $-\frac{\beta_{4i}}{\beta_{3i}}$ and $-\frac{\beta_{5i}}{\beta_{4i}}$ respectively.

## 4 Results and analysis

The results of the research are presented in this part of the report. In the first step of this process, we make an estimate of the descriptive statistics of the variables. Then, we reported the results of the cross-sectional dependence, slope heterogeneity, unit root, and lag selection criteria. Finally, we used panel ARDL model to estimate the empirics.

Descriptive analysis of the variables of interest is shown in Table 1. In this analysis mean, median, maximum value, minimum value, skewness, standard deviation, kurtosis and jarque bera statistics are included. This is generated using panel data from Asian developing countries that span 32 years, from 1990 to 2021. The average female employment is 0.787, with a standard deviation of 0.702. FBS is 2.221 on average, with a standard deviation of 4.568. EDU has a standard deviation of 15.75 and 16.30 mean. 24.47 is the GDP average, with a standard deviation of 3.250. All the variables were shown to be positively skewed. The variables' kurtosis statistic suggests that they are lapto-kurtic (high peak or long-tail) because their values are greater than 3. JQ (Jarque bera) p-value of GDP is larger than 10 %. As a conclusion, we accept the null hypothesis that the data is normally distributed. The Jarque-bera values for all the other variables are also greater than 10 %, leading us to accept the null hypothesis that the data are normally distributed.

According to the available panel data literature, models of panel data are quite likely CSD on the error term [[61], [62], [63]]. Testing for cross-sectional dependence in the selected developing nations is the initial stage. Table 3 displays the results of the cross-sectional dependence test. The statistically relevant test data show that the cross-sections are reliant on each other, which is supported by the CSD test findings. The test results reject the cross-sectional independence null hypothesis for each variable that is addressed. The results demonstrate that shock spreads dramatically throughout developing nations.

**Table 3:** Pesaran CD test (2004) for Cross-Sectional Dependence.

Variables	Statistics	*P*-value
FE	2.550	0.010**
FTS	25.116	0.000***
FBS	34.360	0.000***
SIS	57.610	0.000***
IUI	42.416	0.000***
MCS	57.731	0.000***
EDU	28.618	0.000***
Ln(GDP)	47.683	0.000***

Note: ***, ** and *, represents the level of significance at 1 %, 5 % and 10 % respectively.

Slope heterogeneity test proposed by Ref. [47], which becomes common method in panel data analysis, particularly in productivity and convergence research. It offers a versatile method for capturing individual-specific heterogeneity in panel data models. Table 4 demonstrates that there is a problem of slope heterogeneity. The value of the delta and adjusted delta shows greater slope heterogeneity which means that the relationship between the dependent (e.g., female employment rate) and explanatory variables (digital economy measures, GDP, and EDU) may differ between individual units in the panel.

**Table 4:** Heterogeneity slope test.

Model	Delta	Adjusted Delta
Model 1	12.033	14.238
p-value	0.000***	0.000***

Note: ***, ** and *, represents the level of significance at 1 %, 5 % and 10 % respectively.

The study uses the Cross-Sectionally Augmented IPS [64] test to check the order of integration of the different variables, reported in Table 5. The [64] is a unit root test of second-generation. The findings of the IPS [64] show that FE, MCS, EDU and GDP are integrated of order I (1), whereas FBS and IUI are integrated of order I (0). Hence, for this study panel autoregressive distributive lag (PARDL) technique is applied. Moreover, the result of the test confirms that none of the variables is integrated of order I (2), and hence the current study has used Pooled Mean Group (PMG) technique for the estimation of PARDL, which are reported in table (7).

**Table 5:** Unit root test and lag selection criteria stationarity and order of integration.

Cross-Sectionally Augmented IPS (CIPS, 2007)		
Variables	**Level**	**Order of Integration**
FE	−3.051***	I (1)
FBS	−1.623***	I (0)
IUI	−2.411***	I (0)
MCS	−4.125***	I (1)
EDU	−3.135***	I (1)
GDP	−2.231***	I (1)

Note: ***, ** and *, represents the level of significance at 1 %, 5 % and 10 % respectively.

Table 6 indicates the results of the panel co-integration test. After the existence of CIPS test for stationarity, the current study employs test for co-integration which is [47,65]. The outcomes of the test reject the null hypothesis of no-cointegration and accept the alternative hypothesis of co-integration for the model as stated by the outcomes of the study (see Table 7).

**Table 6:** Test for cointegration second generation panel cointegration test (westerlund, 2005).

Models	Variance Ratio (Statistic)	*P*-value	Cointegration Exists
Model-1			
**FE=f (FTS, FBS, MCS, IUI, SIS, GDP, EDU)**	1.2143	0.0720	Yes

**Table 7:** Results of the PARDL model.

Long Run Equation				
Variables	Coef.	Standard Error	T-stats	Prob
FTS	0.030059	0.005991	5.017693	0.000
FBS	0.211714	0.037906	5.585263	0.000
EDU	0.002154	0.00172	1.252261	0.2119
IUI	−0.01646	0.004251	−3.87148	0.0001
LN_GDP_	0.05785	0.029625	1.9527	0.0522
MCS	0.005467	0.001105	4.94875	0.000
**Short Run Equation**				
Coint.Eq01	−0.16631	0.088636	−1.87631	0.052
D(FE(-1))	−0.348135	0.204932	1.69878	0.0908
D(FE(-2))	0.115176	0.064795	1.777554	0.0569
D(FTS)	0.009527	0.023875	0.399037	0.0403
D(FTS(-1))	0.01522	0.019105	0.79668	0.0965
D(FBS)	0.006704	0.059309	−0.11304	0.9101
D(FBS(-1))	−0.15625	0.102872	−1.51891	0.1303
D(EDU)	0.003558	0.003352	1.061394	0.2897
D(EDU(-1))	0.005387	0.003003	1.793864	0.0743
D(IUI)	0.016438	0.010506	1.564537	0.1192
D(IUI(-1))	−0.01493	0.016532	−0.90289	0.3676
D(LN_GDP_)	0.00544	0.119256	0.04562	0.9637
D(LN_GDP(-1))	0.082847	0.118915	0.696692	0.4868
D(MCS)	0.003947	0.002853	1.383424	0.168
D(MCS(-1))	−0.00055	0.002869	−0.19127	0.8485
D(SIS)	0.003373	0.002482	1.358844	0.1757
D(SIS(-1))	−0.00473	0.005258	−0.89954	0.3694
C	−0.06895	0.075437	−0.91395	0.3618

Above tables shows the long and short run estimates of panel autoregressive distributed lag model which shows the relationship between the female employment (dependent variable) and digital economy (independent variable). ECM, value −0.166 which is a significant and negative effect, demonstrates that the system is converted into long-run equilibrium after a shock. It is evident from the result of this study explain that one year lag in female employment has statistically insignificant as well as negative relation with current female employment. Second lagged female employment (FE) positive correlation with female employment with a coefficient of 0.115 but statistically significant in the short run. Likewise, current-year FBS has a positive correlation with female employment with a coefficient of 0.006, and one-year lagged FBS has a negative association with female employment. In the short run, FTS (Subscriptions for Fixed Telephones) positively and significantly affects on current female employment rates. It shows that in short run a one-unit increment in the FTS tends to increase the current female employment by 0.0095 %. In short run One lag of coefficient of FTS is 0.015 but statistically insignificant impact on female employment. Education has a positive association with dependent variable of female employment. In the short run, digital economy proxies affected female employment positively and negatively reported in the table. But we are not interested in the short-run results our main focus is on the long-run how the digital economy impacts female employment rates in Asian developing countries. All variables are positive and significant impacts on female employment except IUI which is negative but statistically significant. The long-run results show that female employment is positively linked with the FBS and is statistically significant. More specifically that one unit increase in the FBS will tend to increase female employment by 0.21 % each year in the long run. The FTS is positively associated with female employment. Due to one unit increase by the FTS, female employment is also increase by 0.03 %. Result of the MCS and SIS also has a positive influent on female employment. Control variables (education, GDP) also have a significant influence on female employment. If education level increases by 1 % then it tends to increase female employment by 0.002 %. According to the empiric, governments may invest in digital infrastructure and give access to technology to help women to participate in the digital economy. The outcomes of the study are consistent with the previous literature e-i., [66]. They also reported a significant impact of digital economy on female employment. The long- and short-term positive correlation between female employment rates and the digital economy points to a dynamic interplay between labour market dynamics and technological innovation. Short-term opportunities brought about by technology advancements, like flexible work schedules and the need for digital skills, might be blamed for the rise in female employment in the digital economy. Due to the long-term accumulation of human capital and the normalization of digital work practices, women's participation tends to become more entrenched as the digital economy grows. Furthermore, as women advance in their careers in digital domains, they may serve as role models for younger generations of women, strengthening the beneficial association. This long-term trend of benefits highlights how revolutionary the digital economy can be in advancing both economic expansion and gender inclusiveness. The analysis demonstrates that the digital economy has beneficial short- and long-term benefits on female employment, although the type and scale of these effects vary. In the short term, increasing digital adoption and fewer entry barriers may contribute to an increase in job prospects. Over time, sustained improvements are anticipated to result from the cumulative effects of improved digital skills, stronger infrastructure, and greater incorporation of women into the workforce. This shows that, while immediate benefits are significant, long-term policies and investments are required to have long-term impact.

The sensitivity analysis CUSUM and CUSUMsq have been employed to examine the reliability of series. Figs. 1a and 1b show that long and short run parameters are stable and reliable at 5 % significance.

## 5 Conclusion

The main purpose of this research is to examine whether digital economy improves female employment rates in Asian developing nations between 1990 and 2021. According to the data, the digital economy, as evaluated by FBS, FTS, MCS, SIS, and IUI, has a significant impact on female employment in PARDL. The study discovers empirical evidence of a positive relationship between female employment and the digital economy in both the short and long run. The findings also show that a growth in the digital economy leads to a considerable increase in female employment rates in developing countries. Control variables, such as GDP and Education, also have a significant impact on female employment success. Based on the findings, the report recommends that these countries invest in internet infrastructure and encourage e-commerce. It would lead to a major improvement in internet commerce activity, resulting in improved employment opportunities for women in the long run, connecting them to the mainstream workforce, who would otherwise face several hurdles due to cultural and religious formalities. Several measures were used to address potential biases in the study. First, the methodology employs robust statistical techniques such as slope heterogeneity to account for unobserved heterogeneity between countries or regions. Second, the study employs a broad range of control variables (such as education and GDP) to account for other characteristics that may influence female employment. Third, data sources have been carefully chosen to assure dependability and validity, such as using recognized databases like the World Bank or the International Labour Organization. Finally, sensitivity analyses or robustness checks are carried out to ensure that the results are not influenced by particular model parameters or data concerns.

Findings from studies conducted in developing Asian nations may offer insightful information, but because socioeconomic conditions may differ, care must be used when extrapolating the findings to other areas or economic circumstances. In Socio-Economic scenarios: the level of digital infrastructure, educational systems, cultural norms, and gender dynamics varies across different areas and economic situations. These elements may have an impact on how women interact with the digital economy and take part in the labor force. For instance, cultural constraints may make it more difficult for women to pursue higher education or find jobs in the digital industry in some areas, while supporting policies may encourage higher female participation in other areas. Digital Divide: Women's ability to gain from the digital economy is impacted by differences in access to digital technologies and internet connectivity that occur across geographic and economic contexts. Various socio-economic factors, including income inequality, rural-urban divisions, and government regulations, can either intensify or lessen these discrepancies, hence influencing the relationship between female employment and the digital economy in different contexts. Policy Implications: It is important to consider the policy contexts and actions when extrapolating research from developing Asian nations to other regions. To empower women in the digital economy and solve socioeconomic issues, policymakers must customize their approaches. To improve female job possibilities in many situations, for instance, specific investments in digital infrastructure, education, and gender-sensitive policies can be required.

The digital economy has the potential to provide new job opportunities for women in Asia's developing countries. Yet, the benefits of the digital economy may not be properly divided, and women may face unique hurdles in accessing and enjoying these benefits. The following findings have important socioeconomic implications for understanding the relationship between the digital economy and female employment in Asian emerging countries:

**Socio-Economic Development:** By increasing the labor force, encouraging entrepreneurship and innovation, and propelling economic growth, increasing female participation in the digital economy can help to promote socioeconomic development overall. Enhanced work prospects for females in the digital industry may result in increased household earnings, a decline in poverty, and better living conditions.

**Gender Equality**: Giving women more access to education, employment prospects, and decision-making positions via the digital economy advances gender equality. In addition to helping individual women, closing the gender gap in digital literacy and employment promotes an inclusive and egalitarian society.

**Innovation and Skill Development:** Investing in women's entrepreneurship and digital skills can promote technical innovation, increasing the economy's productivity and competitiveness. Through the utilization of women's abilities and viewpoints, communities can cultivate an innovative and creative atmosphere that is advantageous to all constituents.

**Policymakers:** The results can be used by policymakers to create focused initiatives that encourage female involvement in the digital economy. This could entail programs to raise digital literacy, train, and mentor female entrepreneurs, and provide policy frameworks that encourage participation by addressing hurdles that are specific to gender.

In conclusion, the results highlight the importance of using the digital economy to promote gender equality and socioeconomic growth in developing Asian nations. Societies can achieve economic growth, creativity, and social change by empowering women and encouraging their engagement in the digital domain.

## Limitations of the study

By concentrating on female workers who have gotten comparatively less attention in this domain, our research adds to the body of literature already available on digitization and employment. This study have some limitations. First we have facing the data availability issue of the some variables. Secondly this study have missing the gender base analysis. Future studies ought to investigate the effects of the digital economy on female employment across industries and whether digitization leads to the polarization of employment based on gender in different industries. Third this study have missing to analysis the micro factor, in future we can work on it to explore that what are the micro factor that improve female employment.

## Data availability statement

Data will be made available on request.

## CRediT authorship contribution statement

**Riaz Ahmad:** Writing – original draft, Supervision, Software, Resources. **Fatima Sharif:** Formal analysis, Data curation, Conceptualization. **Sareer Ahmad:** Project administration, Methodology, Investigation. **Azeem Gul:** Writing – review & editing, Visualization, Validation. **Zhainagul Abdirasulova Abdirasulovna:** Conceptualization.

## Declaration of competing interest

The authors declare the following financial interests/personal relationships which may be considered as potential competing interests:Riaz Ahmad reports a relationship with Qilu Institute of Technology that includes: employment. NIL If there are other authors, they declare that they have no known competing financial interests or personal relationships that could have appeared to influence the work reported in this paper.
